# Racial Differences in Radium-223 Treatment Response and Adverse Effects in a Small-Cohort, Pilot, Hypothesis-Generating Observational Study

**DOI:** 10.31486/toj.25.0023

**Published:** 2025

**Authors:** Johnny Yang, Mary R. Nittala, Srinivasan Vijayakumar, Vani Vijayakumar

**Affiliations:** ^1^ School of Medicine, University of Mississippi Medical Center, Jackson, MS; ^2^ Department of Radiation Oncology, University of Mississippi Medical Center, Jackson, MS; ^3^ Manipal Academy of Higher Education, Manipal, Karnataka, India; ^4^ Department of Radiology, Ochsner Clinic Foundation, New Orleans, LA

## TO THE EDITOR

Prostate cancer is the second leading cause of cancer-related death for males in the United States and the most common cancer diagnosis for males in the United States and worldwide.^1-3^ Treatments vary for metastatic disease, depending on the extent and type of metastatic spread and castration resistance.^2,4^ Radium-223 (Ra-223) dichloride is a systemic therapy that was approved by the US Food and Drug Administration in 2013 for patients with metastatic castration-resistant prostate cancer^4^ after the ALSYMPCA (Alpharadin in Symptomatic Prostate Cancer) trial showed that Ra-223 improved overall survival and had an appropriate safety profile.^5^

African American males have a significantly higher prostate cancer incidence rate than Caucasian males.^6^ Despite this epidemiological variation, a Veterans Affairs system cohort study reported a lower mortality risk for African Americans in a multivariable analysis of African American and non–African American males (hazard ratio 0.75, 95% CI 0.57 to 0.99; *P*=0.045).^7^ Here, we report our interim observational findings in a small cohort of patients in Mississippi with metastatic castration-resistant prostate cancer who were treated with Ra-223.

Our retrospective, noninterventional, institutional review board–approved cohort study included 9 patients with metastatic castration-resistant prostate cancer who received Ra-223. Of note, Ra-223 is administered in six 1-hour infusion cycles. Prostate cancer was confirmed by biopsy, and bone metastases were confirmed with technetium-99m bone scans. Data collection occurred at treatment initiation, and the review of patient records included baseline variables, medical history, laboratory values, treatment history, disease characteristics, adverse treatment complications, and social variables. The primary study outcome was overall survival measured in months, calculated from the date of diagnosis and stratified by race over the study duration (November 2018 to January 2025). Secondary outcomes were the differences in baseline characteristics. The relationship between categorical variables was assessed using the Pearson chi-square test. The Kaplan-Meier method was used to estimate overall survival rates. Data were analyzed using SPSS Statistics version 29.0.2.0 (IBM Corporation).

Patients had a median age of 77 years (range, 55-87 years) at diagnosis. A higher percentage of patients were Caucasian vs African American (66.7% vs 33.3%), but a greater percentage of patients from the African American group (2 patients of 3, 66.7%) completed all 6 cycles of Ra-223 treatment compared to patients in the Caucasian group (2 patients of 6, 33.3%). A higher percentage of patients in the Caucasian group experienced posttreatment complications compared to patients in the African American group (66.7% vs 33.3%, respectively), but African Americans were more likely to be diagnosed with anemia than Caucasians (100% vs 16.7%, respectively; *P*=0.018).

Univariate analysis by Kaplan-Meier demonstrated that African American patients treated with Ra-223 had a better median overall survival than Caucasian patients (14 months vs 11 months, respectively), but the difference was not statistically significant (*P*=0.359). The bivariate analysis by Kaplan-Meier for overall survival comparing races demonstrated no statistically significant findings.

The interim analysis suggests 2 findings: African Americans had a longer survival time and generally experienced fewer disease-related posttherapy complications compared to Caucasians. Despite suboptimal health outcomes in Mississippi overall,^8,9^ these interim results favor African Americans. We hypothesize that the improved median overall survival for African Americans may be explained by the fewer side effects in that group, so African American patients were therefore more likely to receive more Ra-223 doses than Caucasians.

These observations from the initial interim analysis need confirmation in a large cohort and in institutional studies. Obviously, the major limitation of this study is the small cohort size; hence, the findings must be interpreted with extreme caution. This study should be considered a hypothesis-generating pilot. Efforts to improve our understanding of prostate cancer and metastatic prostate cancer behavior and the response to intervention need broad population-based efforts; such approaches can lead to better optimized personalized/precision cancer care for patients with prostate cancer.^10^

## References

[1] Siegel RL, Miller KD, Wagle NS, Jemal A. Cancer statistics, 2023. CA Cancer J Clin. 2023;73(1):17-48. doi: 10.3322/caac.21763 36633525

[2] Iannantuono GM, Chandran E, Floudas CS, Efficacy and safety of PARP inhibitors in metastatic castration-resistant prostate cancer: a systematic review and meta-analysis of clinical trials. Cancer Treat Rev. 2023;120:102623. doi: 10.1016/j.ctrv.2023.102623 37716332 PMC10591840

[3] Cancer Today. World. World Health Organization: International Agency for Research on Cancer. Accessed April 25, 2025. gco.iarc.who.int/media/globocan/factsheets/populations/900-world-fact-sheet.pdf

[4] Gillette CM, Yette GA, Cramer SD, Graham LS. Management of advanced prostate cancer in the precision oncology era. Cancers (Basel). 2023;15(9):2552. doi: 10.3390/cancers15092552 37174018 PMC10177563

[5] Parker C, Nilsson S, Heinrich D, Alpha emitter radium-223 and survival in metastatic prostate cancer. N Engl J Med. 2013;369(3):213-223. doi: 10.1056/NEJMoa1213755 23863050

[6] U.S. Cancer Statistics Working Group. United States cancer statistics: data visualizations tool. U.S. Department of Health and Human Services, Centers for Disease Control and Prevention and National Cancer Institute. June 2024. Accessed May 1, 2025. gis.cdc.gov/Cancer/USCS/#/AtAGlance/

[7] Zhao H, Howard LE, De Hoedt A, Racial discrepancies in overall survival among men treated with ^223^radium. J Urol. 2020;203(2):331-337. doi: 10.1097/JU.0000000000000524 31479407

[8] Hohl M, Dodd J, Penman A. A retrospective review of the patient population served by the Jackson Free Clinic, a student-run free clinic in Jackson, Mississippi. J Health Care Poor Underserved. 2022;33(1):362-373. doi: 10.1353/hpu.2022.0027 35153226

[9] Jones ML, Vijayakumar S, Nittala MR, Brunson CD. An interdisciplinary perspective on improving cancer care in the state of Mississippi as an example of cancer care improvements in the Global South. Cureus. 2025;17(1):e76865. doi: 10.7759/cureus.76865 39758867 PMC11698381

[10] Yang J, Nittala MR, Velazquez AE, Buddala V, Vijayakumar S. An overview of the use of precision population medicine in cancer care: first of a series. Cureus. 2023;15(4):e37889. doi: 10.7759/cureus.37889 37113463 PMC10129036

